# Human activation-induced deaminase lacks strong replicative strand bias or preference for cytosines in hairpin loops

**DOI:** 10.1093/nar/gkac296

**Published:** 2022-05-07

**Authors:** Ramin Sakhtemani, Madusha L W Perera, Daniel Hübschmann, Reiner Siebert, Michael S Lawrence, Ashok S Bhagwat

**Affiliations:** Department of Chemistry, Wayne State University, Detroit, MI 48202, USA; Department of Pathology, Massachusetts General Hospital, Harvard Medical School, Boston, MA, USA; Broad Institute of Massachusetts Institute of Technology and Harvard University, Cambridge, MA, USA; Department of Chemistry, Wayne State University, Detroit, MI 48202, USA; Molecular Precision Oncology Program, National Center for Tumor Diseases, Heidelberg and German Cancer Research Center, Heidelberg, Germany; Heidelberg Institute for Stem cell Technology and Experimental Medicine, Heidelberg, Germany; German Cancer Consortium, Heidelberg, Germany; Institute of Human Genetics, Ulm University and Ulm University Medical Center, Ulm, Germany; Department of Pathology, Massachusetts General Hospital, Harvard Medical School, Boston, MA, USA; Broad Institute of Massachusetts Institute of Technology and Harvard University, Cambridge, MA, USA; Department of Chemistry, Wayne State University, Detroit, MI 48202, USA; Department of Biochemistry, Microbiology and Immunology, Wayne State University School of Medicine, Detroit, MI 48201, USA

## Abstract

Activation-induced deaminase (AID) is a DNA-cytosine deaminase that mediates maturation of antibodies through somatic hypermutation and class-switch recombination. While it causes mutations in immunoglobulin heavy and light chain genes and strand breaks in the switch regions of the immunoglobulin heavy chain gene, it largely avoids causing such damage in the rest of the genome. To help understand targeting by human AID, we expressed it in repair-deficient *Escherichia coli* and mapped the created uracils in the genomic DNA using uracil pull-down and sequencing, UPD-seq. We found that both AID and the human APOBEC3A preferentially target tRNA genes and transcription start sites, but do not show preference for highly transcribed genes. Unlike A3A, AID did not show a strong replicative strand bias or a preference for hairpin loops. Overlapping uracilation peaks between these enzymes contained binding sites for a protein, FIS, that helps create topological domains in the *E. coli* genome. To confirm whether these findings were relevant to B cells, we examined mutations from lymphoma and leukemia genomes within AID-preferred sequences. These mutations also lacked replicative strand bias or a hairpin loop preference. We propose here a model for how AID avoids causing mutations in the single-stranded DNA found within replication forks.

## Graphical Abstract

**Graphical Abstract:**
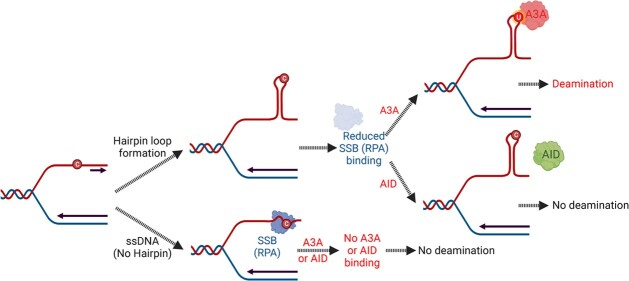
The lack of preference of human AID for cytosines in hairpin loops prevents it from acting at replication forks.

## INTRODUCTION

Activation-induced deaminase (AID) and apolipoprotein B mRNA-editing catalytic polypeptide-like subfamily 3 (APOBEC3) enzymes help protect humans against infections. While AID is active in the adaptive immune response that creates high-affinity antibodies against infectious agents (1,2), the latter group of enzymes are part of the innate immune response against viral infections (3–6). AID is closely related to the APOBEC3s by sequence (7) and all these enzymes deaminate cytosines in single-stranded DNA (ssDNA) to uracil. They cause mutations in the viral or cellular genomes through either replication or error-prone processing of the generated U•G pairs (8). However, there are significant differences between different members of these DNA-cytosine deaminases in terms of expression in different cell types, subcellular localization, preference for base sequence and interactions with other proteins or RNA (5).

In response to an infection, B lymphocytes migrate to the germinal centers in secondary lymphoid organs, express AID and undergo two genetic processes involved in antibody maturation. AID causes base substitution mutations at high frequencies in the immunoglobulin (*IG*) gene variable regions (somatic hypermutation, SHM) and also causes strand breaks in the switch regions of the Ig heavy chain gene, *IGH*, that are processed by the non-homologous end-joining machinery to switch from expression of the IgM isotype to one of the other isotypes, such as IgA (class-switch recombination, CSR; (9,10)). AID also causes SHM in the μ switch region of *IGH* (Sμ; (11)). Transcription of the *IG* genes is required for efficient SHM and CSR (10,12). In B cell cancers, AID is frequently expressed constitutively (13–16), and is thought to promote mutations in non-*IG* genes (17,18) and chromosomal translocations (19). The principal unanswered questions regarding the role of AID in antibody maturation include (A) How AID exploits transcription of the *IG* genes to find cytosines for deamination; (B) How it avoids introducing uracils in DNA outside the Ig genes during normal antibody maturation; (C) Whether it can deaminate cytosines in the single-stranded stretches of DNA within the replication forks and (D) What are the secondary structures in DNA that are preferred or disfavored by AID.

Regarding the last two points, past work has better defined the preferences of APOBEC3s. Using yeast (20) or *Escherichia coli* (21,22) model systems it was shown that APOBEC3A (A3A), APOBEC3B (A3B) and APOBEC3G carboxy-terminal domain (A3G-CTD) preferentially target cytosines in the lagging-strand template (LGST) over those in the leading-strand template (LDST). This strand bias is mirrored in the distribution of mutations found in cancer genomes that are attributed to these mutations (APOBEC signature mutations; (23–25)). Additionally, A3A prefers cytosines in the loops within hairpins over many other structures (22,26–28), while A3B and A3H may show lesser preference for hairpin loops (22,27,28). Furthermore, the mammalian single-strand DNA binding protein, replication protein A (RPA), inhibits the activity of A3A on linear DNA substrates, but does so inefficiently for a hairpin loop substrate probably because of poor binding of RPA to the hairpin (26,29). APOBEC3G (A3G) activity on a linear substrate is also inhibited by RPA (30).

Bubbles within duplex DNA are considered to be similar to the R-loops that are known to form in the switch regions of *IG* genes (31,32), and characterization of human AID suggested that it prefers cytosines within DNA bubbles compared to linear DNAs (33–35). Based on *in vitro* studies of DNA bubbles and hairpin loops ranging in size from 1 nt to 13 nt, Larijani and Martin concluded that AID deaminates cytosines in DNA bubble structures much faster than those in hairpin loops (35). Other studies have suggested that AID prefers cytosines in more elaborate frameworks such as Y-structures and G-quadruplexes in DNA (36,37). It is not known whether AID prefers cytosines in LGST over those in LDST, and the role played by RPA in deamination by AID is controversial. While one report found that RPA recruits AID for transcription-dependent cytosine deamination in an *in vitro* transcription/deamination assay (38), a later report contradicted this finding (39).

To clarify the role played by transcription, replication and DNA secondary structure in the ability of AID to deaminate cytosines, we applied UPD-seq, a technique by which uracil-containing DNA fragments are pulled-down and sequenced (22,28), to the genome of uracil repair-deficient *Escherichia coli* expressing a form of the human AID that is more soluble than wild-type AID (37). This full-length mutant contains five amino acid changes (Supplementary Figure S1A), and is called AID.crystal or AID.cry because it was used for the determination of its structure using X-ray crystallography (37). We chose it over wild-type AID because AID.cry promotes robust CSR, is mutagenic in *E. coli* and its interaction with different DNA substrates has been studied biochemically. It contains replacement of five residues which lie on the surface of the protein that is on the other side of the enzyme active site (37). Although it introduces three negatively charged residues on the protein surface (F42E, R131E and F145E) (37) that may weaken sequence non-specific interactions of AID with DNA backbone, King *et al.* (40) found that the structure of AID.cry is in very good agreement with WT AID structure predicted from computational and biochemical studies. UPD-seq allowed us to examine the substrate preferences of AID.cry across a wide variety of potential hairpin loop sequences and between the two strands within the replication fork. We then examined the somatic mutations attributable to AID in human hematological tumors to determine whether the pattern of distribution of these mutations was similar to the distribution of uracils created by AID.cry in *E. coli*.

## MATERIALS AND METHODS

### Bacterial strains and plasmid constructs

*Escherichia coli* K12 strain BH214 (*ung mug*::miniTn10 *dcm6 thr1 hisG4 leuB6 rpsL ara14 supE44 lacY1 tonA31 tsx78 galK2 galE2 xyl5 thi1 mtl1;* λ DE3 = sBamHIo ΔEcoRI-B *int::(lacI::*PlacUV5::T7 gene1) i21 Δ*nin5*) was obtained from Dr. William Franklin (Albert Einstein College of Medicine). A clone of the human AID variant, AID.cry (37), in the bacterial expression vector pTrc99A was obtained from Dr Hao Wu (Harvard University). AID.cry gene was amplified using PCR and cloned into vector plasmid pASK-IBA5C (IBA Lifesciences) as an EcoRI–BamHI fragment. Primers used for the cloning are listed in Supplementary Table S1 and all clones were validated using Sanger sequencing (DNA sequencing core, University of Michigan).

### Cell growth and mutational assays

Independent colonies of BH214 cells containing different plasmids were grown overnight at 37°C followed by a 100-fold dilution in Luria-Bertani media with chloramphenicol (35 μg/ml) and incubated in a shaker at 37°C for 2 h. To induce AID expression, cells were diluted again 10-fold in LB containing the antibiotic and 0.5 μg/ml anhydrotetracycline (Cayman Chemicals). The cultures were grown until OD_600_ reached 0.8–0.9. Appropriate dilutions were spread on plates with chloramphenicol alone or with rifampicin (100 μg/ml) and chloramphenicol to determine the frequency of rifampicin-resistant cells (= number of colony forming units per milliliter on rifampicin plus chloramphenicol plates/number of colony forming units per milliliter on chloramphenicol plates). The remaining cultures were harvested and used for genomic DNA extraction.

### Preparation of DNA for uracil quantification and UPD-seq

The cells were broken by incubating them in a solution containing 1× TE (10 mM Tris–HCl (pH 8), 1 mM EDTA), 1% SDS and Proteinase K (2 mg/ml) at 37°C for 1 h. The resulting viscous solution was aspirated several times using 26-gauge needle to break up the DNA. Proteins were removed from the mixture using extraction with phenol: chloroform (1:1), and the DNA was precipitated by the addition of 0.2 volumes of sodium acetate (3 M) and two volumes of ethanol followed by the incubation at –20°C for 30 min. DNA was harvested by centrifugation at 25 000 g for 10 min and washed with 70% ethanol. It was dried and dissolved in 1× TE. To remove RNA, the DNA preparation was treated with 2 μg/ml of RNase A at 37°C for 1 h. This DNA was again precipitated using ethanol and dissolved in 1× TE.

For UPD-seq, the genomic DNA was broken up using Covaris S2 sonicator (Applied Genomics Technology Center WSU) to produce ∼500 bp fragments. For uracil quantification, 5 μg of genomic DNA from each sample was digested with HaeIII (NEB) and purified with phenol: chloroform extraction followed by ethanol precipitation. In both cases, the fragmented DNA was incubated with AA7 (*O*-allyl-hydroxylamine hydrochloride, Sigma Aldrich, 10 mM final concentration) at 37°C for 1 h to block the pre-existing abasic sites (41). The DNA was further incubated with *E. coli* uracil DNA-glycosylase (Ung) at 37°C for 30 min to excise the uracils and then incubated with 5 mM AA6 (to quantify uracils (41)or 10 mM ssARP for 1 h at 37°C (to label the resulting abasic sites with biotin (22)). AA6 and ssARP are previously described alkoxyamines that react with abasic sites created by Ung (22,42).

### Quantification of genomic uracils

AA6 tagged DNA was labeled with DBCO-Cy5 (1.7 μM) under Cu-free conditions by shaking the reaction mixture for 2 h at 37°C in dark. Labeled DNA was purified using DNA Clean and Concentrator kit (Zymo research). Fluorescently labeled DNA was transferred on to a positively charged zeta probe membrane (Bio Rad) using a dot blot apparatus (Bio-Rad) and the membrane was scanned using a Typhoon 9210 phosphor imager (GE Healthcare). Images were analyzed using the ImageJ software.

### UPD-seq of *E. coli* genomic DNA

The DNA labeled with ssARP was bound to the Dynabeads MyOne Streptavidin C1 (Invitrogen), the beads were washed with the manufacturer recommended 2× DNA binding and wash buffer (B&W buffer) and were separated from the solution on a magnetic stand (DynaMag, Invitrogen). The supernatant containing the unbound DNA was removed, the beads were washed with 1× B&W buffer, and resuspended in 1× TE. The bound DNA was released from beads by incubating with 100 mM dithiothreitol for 10 min at 37°C. Beads were placed on the magnetic stand, and the supernatant which contained the eluted DNA was collected. DNA was concentrated using ethanol precipitation.

The pulled-down DNA was used for DNA library preparation using Illumina TruSeq nano kit (Illumina). All the libraries were pooled in equimolar quantities for multiplexed sequencing and sequenced on Illumina MiSeq platform (Michigan State University). The sequencing was performed in a 2× 150 bp paired-end format using a MiSeq v2 300 cycle reagent cartridge. Base calling was done by Illumina Real Time Analysis (RTA) v1.18.54 and output of RTA was demultiplexed and converted to FastQ format using Illumina Bcl2fastq v2.19.1. The list of fastq files and their accession numbers are provided in Supplementary Table S3.

### Sequence alignment and analysis

DNA sequence alignment and analysis was performed using LINUX-based software available at the High-Performance Computing Grid at Wayne State University. Sequencing reads that were mapped to the plasmid pASK-IBA5C in BH214 were removed and the remaining sequencing reads were aligned to the *E. coli* reference sequence using BWA (version 0.7.12) (43). Sequencing reads containing plasmid sequences were removed and Samtools (Version 1.9) (44) was used to re-format, index and sort the alignment file and extract the depth of coverage at each genomic position.

A bash script was written to filter the sequence reads that do not have any mismatch at a G:C reference position. The alignment file was filtered to remove all perfect matches and to select reads that had a mismatch at a reference C or G position. This filtered bam file was then used to extract depth of coverage across the genome. The unfiltered alignment file was used as the input to extract the nucleotide composition of reads at each genomic position by bam-readcount (https://github.com/genome/bam-readcount).

### Identification of uracilation peaks

NDC (Normalized differential coverage) was used previously to map uracilation peaks created by A3A (22). We improved the NDC algorithm to eliminate local as well as global fluctuations in depth of coverage. A local moving average (*mav*) in a window of 120 bp was calculated on the depth of coverage and a regional moving average window of 100 kb was also calculated, and these were used to normalize the depth of coverage. The difference in the depth of coverage (DOC) between the sample and empty vector (EV) library was used to define NDC2 according to the equation below:

$$\begin{equation*}NDC2\; = \;\frac{{mav\;\left( {sample\;DOC,\;120\;bp} \right)\;}}{{mav\;\left( {sample\;DOC,\;100\;kbp} \right)}} - \frac{{mav\;\left( {EV\;DOC,120\;bp} \right)\;}}{{mav\;\left( {EV\;DOC,\;100\;kbp} \right)}}\end{equation*}$$

The sizes of the two moving windows were optimized to maximize the signal-to-noise ratio.

R software for statistical computing (version 3.4.1; https://www.r-project.org/) was used to the calculate NDC2 and make the barcode plots. The uracilation peaks were defined as the regions where NDC2 signal was above 5σ (5 times the standard deviation) of NDC2 across the genome. The genes overlapping the peaks were identified by performing BLAST alignment of the sequences of the peaks with *E. coli* K-12 MG1655 sequence and the transcription start sites for the genes were obtained from the EcoCyc database (45). The peaks detected for AID.cry were correlated with peaks found for A3A using the R package, GenometriCorr (46).

### Calculation of uracilation index within specific sequences and hairpin loops

Uracilation index (UI) for any sequence (e.g. WRCY) in the genome was defined by the following equation:

$$\begin{eqnarray*}&&Uracilation\;Index\;( {UI} ) \nonumber\\ &&= \frac{{\sum ( {\frac{{Number\;of\;C:G\;to\;T:A\;changes\;at\;a\;specific\;sequence\;}}{{Depth\;of\;coverage\;at\;that\;position}}} )}}{{Number\;of\;occurences\;of\;the\;sequence\;in\;the\;genome}} \times {10^3},\end{eqnarray*}$$

where the summation is performed over all the sequences of that type in the genome.

The BH214 reference genome was scanned for potential hairpin-forming sequences using the ApoHP tool as described previously (47) and UI was calculated for the hairpin-forming and non-hairpin sequence sets. Software packages ggplot2 (48), and Biorender (biorender.com) were used to generate the figures. To estimate the statistical robustness of the UI comparisons, BH214 genome was randomly sampled into three equal subsets and UIs were calculated for each subset separately. The genomic subsets had roughly the same numbers of representative positions across different variables such as GC content, hairpins, and replicative and transcriptional strands. These measurements were taken as separate data points to calculate the error bars in Figures 3 and 4.

### Replicative and transcriptional strand bias in the *E. coli* genome

Based on the origin of replication and termination point within the *E. coli* genome (45) the reference sequence was divided into LGST and LDST sequences. The UI was calculated separately for the two sequence sets based upon whether the cytosine that was converted to thymine was in LGST or LDST. Similarly, the cytosines in the genome were separated into two subsets based on whether they were in the transcribed strand (Tx-) or the non-transcribed strand (Tx+) of the gene and UI for each subset was calculated (24).

### Analysis of B cell tumor genome sequences

For the AID cohort, we selected a subset of 65 samples among the dataset of 3004 samples subjected to whole genome sequencing (WGS) that were previously analyzed (28). This subset had various hematological tumor types including acute lymphoblastic leukemia (ALL), chronic lymphocytic leukemia (CLL), diffuse large B-cell lymphoma (DLBCL), and multiple myeloma (MM) where fractions of somatic mutations attributed to APOBEC signature by the NMF analysis (*k* = 8) was <5%. In addition, data from WGS of 59 mantle cell lymphoma (MCL) tumors were taken from Nadeu *et al.* (49) and WGS data of a total of 213 germinal center B-cell derived lymphoma including follicular lymphoma (FL), DLBCL and Burkitt lymphoma (BL) were also added from the ICGC MMML-Seq consortium (50,51). The latter two WGS data sets were also subjected to NMF analysis (*k* = 8) to ensure <5% of somatic mutations could be attributed to the APOBEC mutational signatures.

For the APOBEC cohort, a subset of 109 patients was selected from the 3004 WGS patients (28), where more than 50% of mutations were attributed to the APOBEC mutational signature by NMF analysis (*k* = 8). We also excluded patients where >10% of the mutations were attributed to other hypermutational sources such as microsatellite instability, smoking-associated mutagenesis, ultraviolet light and mutant DNA polymerase ϵ. When the AID and A3A cohorts were combined with the TCGA mutation dataset, and NMF analysis (*k* = 10) was run, we were able to detect the COSMIC AID signature SBS84 (52) in >92% of the AID tumors (cutoff equal to 10% of tumor's total mutations). This signature was virtually absent among the A3A tumors with only one sample showing >10% AID signature.

After passing the somatic base substitution mutation data through a Panel of Normals filter for quality control (53), there were a total of 2 213 649 mutations in the AID cohort, and the median number of mutations per patient was 3929 (range 433–117 780). The APOBEC cohort had a total of 2 086 734 mutations with a median of 9832 mutations per patient (range 960–90 211).

All the tumor mutation analysis was performed in MATLAB (Version 9.1.0.441655 (R2016b)) and plots were generated using R (version 4.0.2 (2020-06-22)). Computational analysis was performed on MGB’s ERISOne Linux Cluster or Wayne State University's High-performance computing grid.

### Identification of tumor mutations within hairpins

The human reference genome (hg19) was scanned for potential hairpin-forming sequences using the ApoHP tool (28). Unless noted otherwise, a stem strength (SS) of 12 was used as the threshold to consider a sequence as a hairpin. Hairpin sequences with loop sizes of 3–8 nt were selected and the number of mutations from each cohort at these positions was counted. The mutation counts were normalized to the number of representative hairpins in the genome as well as the overall mutation rate of the cohort.

### Calculation of replicative and transcriptional strand bias in tumor mutations

Mutations at C:G pairs in different nucleotide contexts (NC, TC or WRCY) were counted in the early-replicating regions and the replicative strand bias was calculated as the normalized ratio of mutations at C:G positions where the targeted C was on the LGST to those mutations where the C was on the LDST. The replicative strands were defined as described by Haradhvala *et al.* (24) based on data from Chen *et al.* (54). Mutations were normalized to the baseline rate of mutation in each cohort.

Similarly, mutations in the transcribed regions were counted and the transcriptional strand bias was calculated as the ratio of C:G mutations where C is on the non-transcribed (coding) strand to where C is on the transcribed strand (24). Mutations were normalized to the number of such C:G positions throughout the genome and the overall mutation rate in each cohort.

To determine the relationship between levels of transcriptions of genes and mutations, the genes were divided into 10 bins based on the average expression levels across 91 cancer cell lines from the Cancer Cell Line Encyclopedia. Mutation counts were calculated in each cohort, at different nucleotide contexts. The mutation counts were normalized to the number of C:G positions in each bin as well as the mutation rate in the cohort. The 95% confidence intervals were estimated from, n, the number of mutations and, *N*, the number of representative genomic positions.

## RESULTS

### General features of AID.cry uracilome in *Escherichia coli*

Both the wild-type (WT) human AID and AID.crystal (AID.cry) were expressed in repair-defective *E. coli* cells from a doxycycline-inducible promoter (Supplementary Figure S1A and B) and their mutagenicity was determined using the rifampicin-resistance (Rif^R^) assay. AID.cry caused thirteen-fold more Rif^R^ mutants than WT AID, and while the background levels of uracils in *E. coli* DNA are likely to be due to the utilization of dUTP during replication (55), the expression of AID.cry resulted in significantly higher accumulation of uracils in the cellular genome compared to WT AID (Supplementary Figure S2A and B). The catalytic activity of AID.cry was required for these increases and the expression of the E58A mutant of AID.cry did not cause an increase in Rif^R^ frequency. These results confirm and extend the results reported by Qiao *et al.* (37) and suggested to us that it would be easier to detect and map uracils created by AID.cry than WT AID.

**Figure 1.**
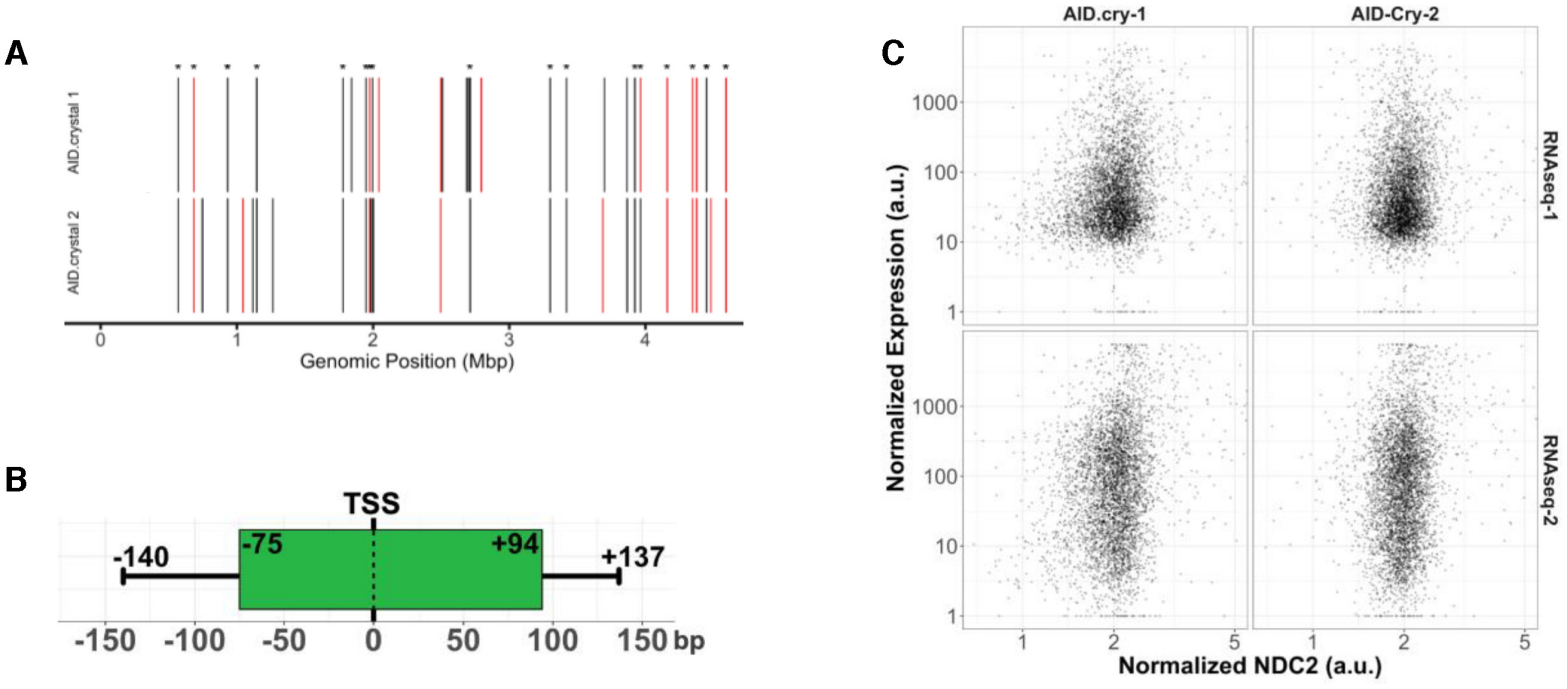
Features of uracilation peaks of AID.cry. (**A**) Uracilation Peaks detected by normalized differential coverage (NDC2) analysis comparing UPD-seq of AID.cry with EV are shown. The peaks that overlap with tRNA genes are marked in red, while protein coding genes are marked in black. The results from two independent experiments are shown. Asterisks denote common peaks. (**B**) The relative position of uracilation peaks overlapping a TSS; median and third quartile distances of boundary positions of peaks to and from the TSS position is shown as the boxplot and whiskers. (**C**) Scatter plots show normalized RNA expression from two different studies and normalized NDC2 values from two replicates at each gene. Values are normalized over each sample as well as the length of the genes. Both X and Y axis are on a logarithmic scale.

DNA from cells expressing AID.cry was subjected to UPD-seq and the results were compared to the results from cells containing EV. The data were analyzed using a modified form of the previously described normalized differential coverage algorithm, NDC (22). The new version, NDC2, reduces the noise in the depth of coverage and increases the signal-to-noise ratio compared to the original NDC algorithm. As a consequence, it detects a greater number of uracilation peaks using the same stringent statistical criterion, i.e. differential depth of coverage five times standard deviation above the mean (5σ, Supplementary Figure S3). In two independent experiments with AID.cry, NDC2 respectively identified 35 and 37 peaks of which 17 were common to both the datasets (Figure 1A). The reasons why some of the peaks between the two datasets do not overlap include experiment-to-experiment variation, but also the high threshold used to define a peak (5σ). If the threshold were lowered, many of the currently unshared peaks between the two datasets would overlap (analysis not shown). The uracilation peaks created by AID.cry covered only about 8000 bp of the genome (0.2%), and were highly enriched in tRNA genes. When the two datasets were combined, 27 of 87 tRNA genes in the *E. coli* genome overlapped with uracilation peaks (Supplementary table S2).

Although most peaks were close to transcription start sites (TSS) and about half of them overlapped TSS (Figure 1B), there was no correlation between transcription levels of genes and the average NDC2 values for the genes (Figure 1C). Finally, when all the genes were considered together, the transcriptional strand bias in uracilation for the AID.cry datasets (1.03) was similar to that for EV (1.04; Supplementary Figure S4A).

**Figure 2.**
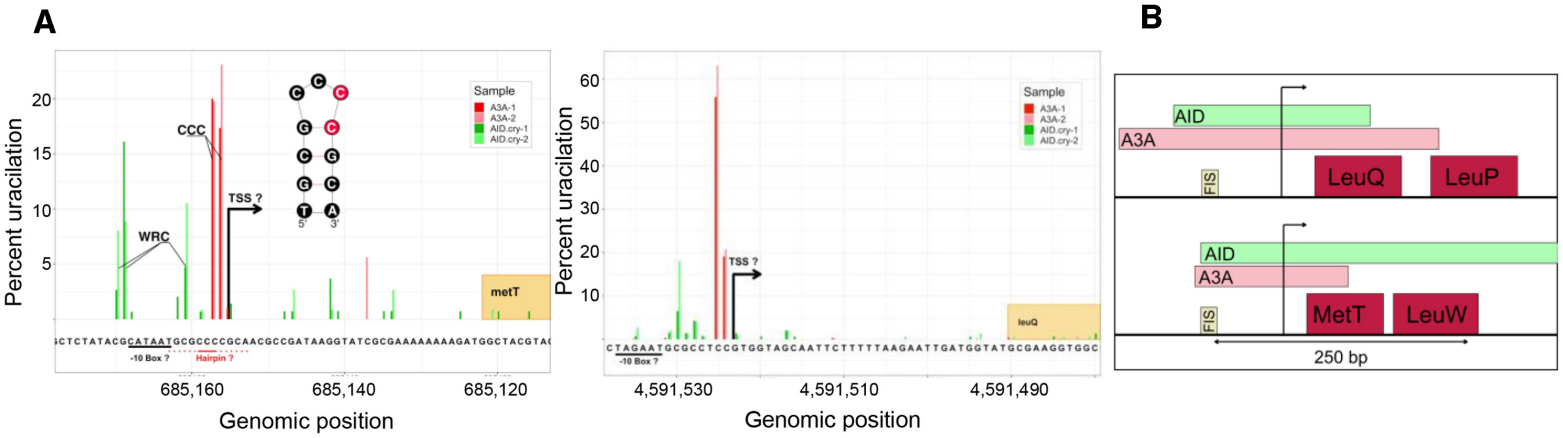
The two genes common within AID.cry and A3A uracilation peaks. (**A**) The percentage of uracilation at position of the sequence surrounding the TSS of *metT* and *leuQ* genes. The uracilation due to A3A is shown in red, while uracilation due to AID.cry is shown in green. Results from both the data sets for each deaminase are shown. The potential hairpin loop overlapping TSS of *metT* is drawn as an inset. (**B**) The presence of a FIS-binding site near the uracilation peaks created by AID.cry and A3A. The arrow represents TSS.

These features of uracilation created by AID.cry were similar to those reported previously for A3A (22). A3A also creates a limited number of uracilation peaks that overlap a large fraction of *E. coli* tRNA genes and TSS positions, but they are not significantly correlated with high transcription (22). Despite these similarities, only two uracilation peaks generated by AID.cry and A3A overlapped, probably because of different sequence specificities of the two enzymes (WRCY and TC, respectively). Regardless, this was a statistically significant overlap (*P*-value = 3.5 × 10^–6^). Both these genes, *metT* and *leuQ*, encode tRNAs and the uracilation peaks overlapped putative transcription initiation signals for the two genes (Figure 2). As expected, the cytosines that were most frequently targeted within the peaks were within WRC for AID, and TC or CC for A3A (Figure 2A). Interestingly, the cytosines targeted by A3A upstream of the expected TSS for the *metT* gene were within a predicted hairpin loop (Figure 2A), and the 3' cytosine in a CCC loop sequence is one of the preferred targets of A3A for trinucleotide loops (22,28).

The genomic region where these overlapping peaks due to AID.cry and A3A lie contains a binding site for the *E. coli* nucleoid-associated protein called factor for inversion stimulation, (FIS; Figure 2B). FIS is a multi-functional regulator with nearly 900 DNA-binding sites (56) and is involved in replication, site-specific recombination, transposition, transcription initiation and chromosome boundary formation (57). While it is clear that not every DNA binding site for FIS is associated with a uracilation peak, there is statistically significant correlation between the occurrence of the peaks and FIS binding sites. When positions of FIS-binding sites in the genome are compared with the positions of peaks in the AID.cry and A3A uracilomes, they are correlated with *P*-values of respectively 3.7 × 10^–8^ and 0.026.

### AID.cry does not have a strong replicative strand bias

Non-enzymatic water-mediated deamination of cytosines in *E. coli* causes a small strand bias in C-to-T mutations in favor of the LGST over the LDST (21,58) and we previously showed that A3A increases this bias (22). A similar strand bias in C-to-T mutations was also reported when A3A and A3B were expressed in yeast (20). To determine whether AID also increases the intrinsic replicative strand bias in uracilation we determined the C:G-to-T:A changes in UPD-seq data from AID.cry, A3A and EV datasets at NC, TC and WRCY sequence contexts across the genome and identified cytosine deamination in either the LDST or LGST in one of the two replichores (Supplementary Figure S4B).

**Figure 3.**
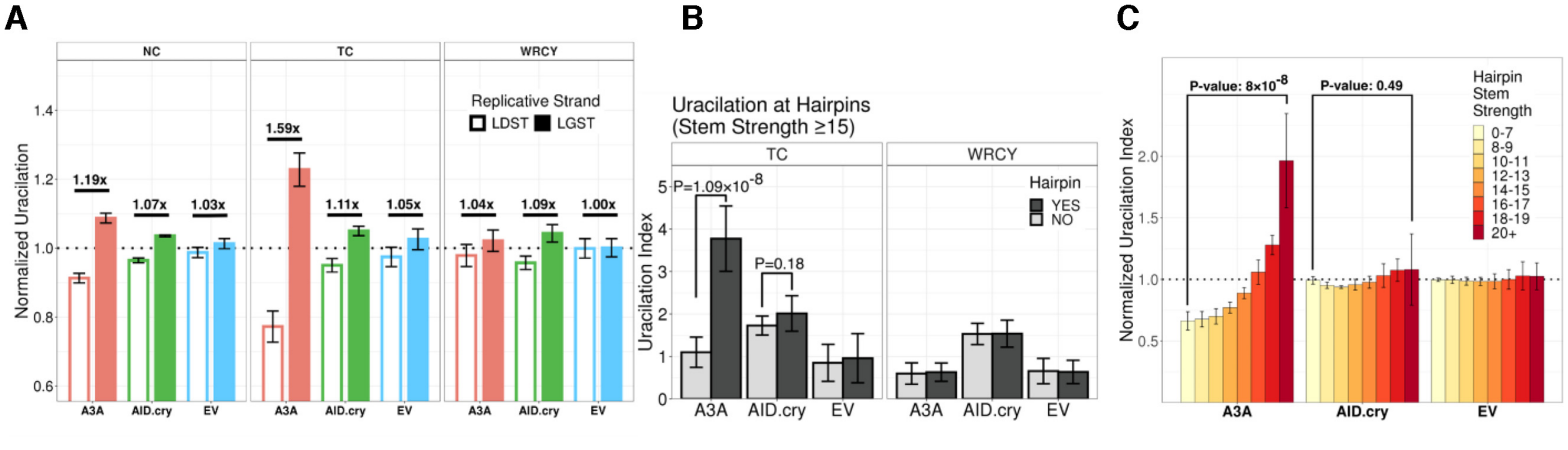
Replicative strand bias in uracilation created by AID.cry and A3A. (**A**) Uracilation index is calculated in each sample for cytosines on the replicative strands LDST and LGST at different sequence contexts, NC, TC and WRCY. These values are normalized for each sample. (**B**) Uracilation index in hairpin forming sequences (potential hairpin stem strength ≥ 15) or not, at TC or WRCY context. (**C**) Uracilation indices from different samples are calculated at potential hairpin forming sequences with different stem strengths. Error bars show standard deviations.

All three uracilation profiles showed the same replicative strand bias across the *E. coli* genome; more uracilation in the LGST compared to LDST, but the magnitude of the strand bias was much higher for A3A than the other two experimental samples (Supplementary Figure S4C). When the duplicate UPD-seq datasets for each condition (EV, A3A or AID.cry) were combined, EV datasets had a weak bias in favor of LGST and the LGST/LDST ratio was <1.1 for all three sequence contexts (Figure 3A). A3A showed much higher strand bias in NC and TC sequences (ratio ≈1.2 and ≈1.6, respectively), but not in the WRCY context (ratio < 1.1; Figure 3A). In contrast, AID.cry caused a weak bias in favor of LGST in all three sequence contexts (ratio ≤ 1.1; Figure 3A). These data suggest that AID.cry deaminates cytosines in WRCY sequences within LGST only slightly more frequently than the non-enzymatic deamination processes.

### AID does not have strong preference for hairpin loops

When the uracilation index (UI) of cytosines in predicted hairpin loops in the *E. coli* genome was compared with the UI of non-hairpin cytosines, the pattern for AID.cry was very different from A3A. Unlike A3A, which strongly prefers cytosines in hairpin loops, UI for cytosines in hairpins and non-hairpins was nearly the same in cells expressing AID.cry (Figure 3B). Furthermore, the slight preference for cytosines in hairpin loops seen in the AID.cry data was similar to the EV control suggesting that AID.cry does not prefer cytosines in hairpin loops (Figure 3B). When the hairpins were separated based on stem strength (SS), UI profile of AID.cry uracilome was mostly similar to that of EV except at the highest SS values. At SS values above 20, there was a small increase in UI, but this increase was not statistically significant (Figure 3C). Together these data show that AID lacks a preference for the predicted hairpins within the *E. coli* genome.

### AID loop 1 does not eliminate A3A preference for hairpin loops or LGST

Recent studies have suggested that loop 1 of A3A, which is shortest among all the AID/APOBEC enzymes, plays a key role in its high activity by making its active site more open (59,60). We wondered whether the presence of a larger loop 1 sequence in AID (Supplementary Figure S5A) could explain the enzyme's lack of preference for the more structured hairpin loop sequences. To test this, we replaced the loop 1 of A3A with loop 1 of AID and found that this A3A-AIDL1 mutant was more mutagenic in *E. coli* than A3A (Supplementary Figure S5B).

**Figure 4.**
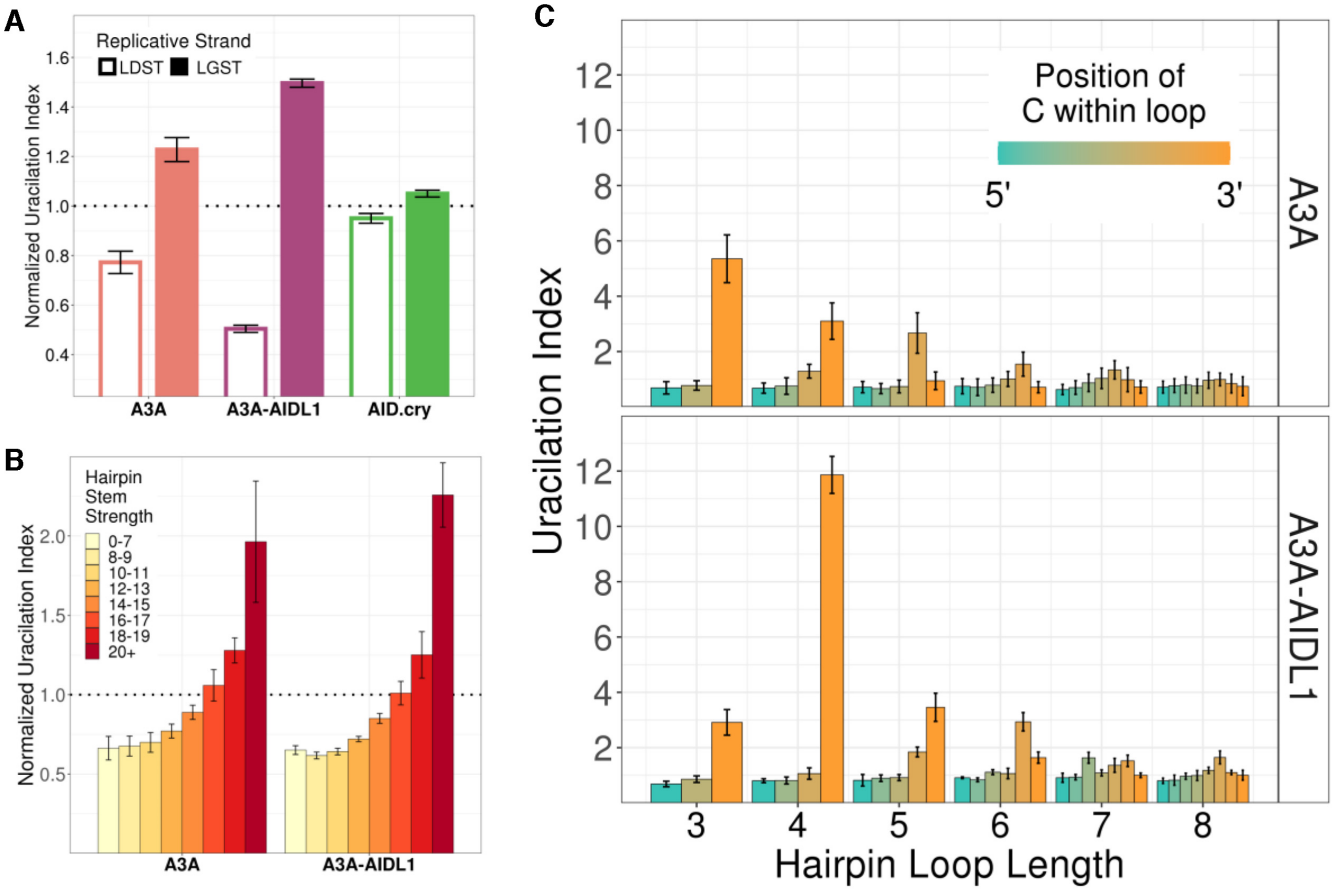
Hairpin loop preferences of A3A-AIDL1 mutant. (**A**) Uracilation index of A3A, A3A-AIDL1 and AID.cry samples at TC sequence context with respect to the replicative strand. (**B**) Uracilation index of A3A and A3A-AIDL1 samples are shown at potential hairpin sequences with different stem strengths. (**C**) Uracilation index of A3A and A3A-AIDL1 are shown as a function of loop length. For each loop length, the position of the cytosine within the loop is shown as a separate bar. Only hairpins with stem strength ≥12 are used. Error bars show standard deviations.

When UPD-seq was performed on *E. coli* expressing the A3A-AIDL1 mutant, the deaminase was still found to prefer cytosines in the LGST (Figure 4A) and hairpin loops and the UI increased with increasing stem strength (Figure 4B). Like A3A, A3A-AIDL1 also showed a preference for short hairpin loops and for cytosines at or near the 3' end of the loops (Figure 4C). Surprisingly, in contrast with the preference of WT A3A for 3 nt loops, A3A-AIDL1 preferred 4 nt loops over 3 nt loops (Figure 4C). This shows that the replacement of loop 1 of A3A with the corresponding sequence from AID does not cause the former enzyme to lose its replicative strand bias or preference for short hairpin loops, but does change the preference of the enzyme for 3 nt loops.

### AID signature somatic mutations in tumors also lack replicative strand bias or preference for hairpins

To determine whether the lack of replicative strand bias and the absence of hairpin loop preference in the *E. coli* data for AID.cry was consistent with preferences of AID in the human genome, we examined mutations found in whole genome sequencing of leukemia and lymphoma genomes. The overwhelming majority of these mutations lie outside the *IG* genes and hence they are unlikely to be affected by processes that specifically shape mutations in the *IG* loci. The base substitution mutations at the C:G pairs in the TC and WRCY sequences within the remaining tumors were mapped to the genome and the mutations within predicted hairpin loops and replicative template strands were identified. These mutations were also mapped to early-replicating regions of the genome and classified according to whether the cytosine was in the LGST or LDST. A similar analysis was performed on tumors that had overwhelmingly high proportion of APOBEC signature mutations.

**Figure 5.**
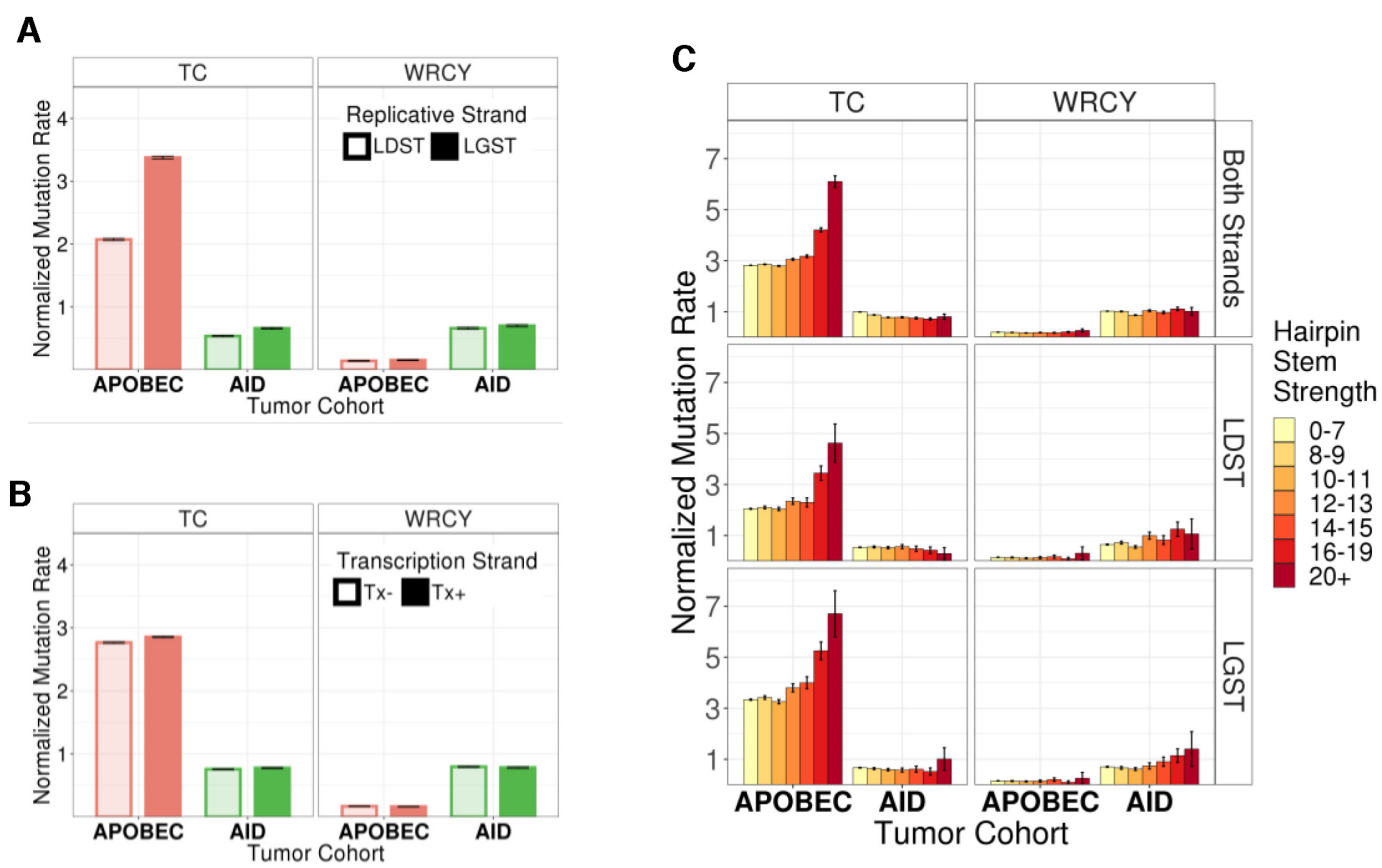
Properties of somatic mutations in the APOBEC and AID tumor cohort. (**A**) Replicative strand bias. The mutation rates were calculated in early replicating regions in LDST and LGST strands for both the APOBEC and AID tumor cohorts at TC and WRCY contexts. (**B**) Transcriptional strand bias. Mutation rates were calculated in the two transcriptional strands at TC and WRCY contexts. Tx+ and Tx– positions are the cytosines on the non-transcribed and transcribed strand during transcription, respectively. (**C**) Normalized mutation rates for APOBEC and AID tumor cohorts within different hairpins were binned based on their predicted stem strength. The sequence context of the mutations is at the top of each column and the mutations in each row are both strands (top row), LDST (middle row) or LGST (bottom row). Error bars in all the panels are 95% confidence intervals.

There was a large difference between the AID and APOBEC signature mutations in terms of their replicative strand bias. While the APOBEC signature mutations in TC sequences had a strong bias in favor of the LGST, AID signature mutations showed only a very modest preference for LGST in these sequences (Figure 5A). Importantly, neither tumor cohort showed a replicative strand bias within the WRCY sequence context preferred by AID (Figure 5A). It should be noted that both AID and APOBEC signature tumor mutations lack a strong transcriptional strand bias ((24) and Figure 5B).

The mutations in the AID cohort also did not prefer hairpin loops. When mutations in the loops were binned based on the strength of their stems, the normalized mutation rate for mutations within the AID cohort at TC and WRCY sequences did not change as a function of stem strength. This was true regardless of whether the sequence was in the LGST or LDST (Figure 5C). In contrast, the APOBEC cohort mutations within TC sequences, but not in WRCY sequences, increased with stem strength when these sequences were in the loops. APOBEC cohort mutations showed this preference in both the replicative templates with the higher mutation rates when the TC was in the LGST rather than the LDST (Figure 5C). Thus the tumor mutation data are consistent with the results from *E. coli* UPD-seq.

## DISCUSSION

### Similarities between the A3A and AID.cry uracilomes

We have shown here that like A3A, a functional mutant of human AID, AID.cry, selectively deaminates cytosines in some parts of the *E. coli* genome much more frequently than others. These deaminations create peaks in the uracilome that are reproducible and have some of the same properties as the uracilation peaks generated by A3A. In both the cases, the peaks overlap tRNA genes at frequencies far exceeding what would be expected based on the number of tRNA genes in the genome and the number of bases covered by these genes. However, most of the uracilation peaks created by A3A and AID.cry did not overlap. It is possible that this is due to the different base-sequence preferences of the two enzymes (TC and WRC, respectively), but may also reflect other factors such as DNA secondary structures or the ability of these enzymes to compete for binding to ssDNA with the single-strand DNA-binding protein, SSB.

A majority of AID.cry and A3A peaks overlapped TSS of genes (Figure 1B), but there was no direct correlation between levels of transcription of genes and their uracilation (Figure 1C). Even when transcription levels of genes with high NDC2 values were plotted as a function of the NDC2, only a modest correlation between the two values was found at high NDC2 values for one of the transcription data sets, but not the other (Supplementary Figure S6). These data are consistent with earlier work in yeast where mutations caused by AID were mapped (61). In that study, 57% of the mutations caused by AID were in the promoter region defined as 500 base pairs upstream and 50 base pairs downstream of the TSS (61). We also found that, like A3A, the uracilation peaks due to AID.cry do not overlap with previously determined R-loop containing regions in the *E. coli* genome identified using the S9.6 antibody [Supplementary Fig S7; (22)]. These data suggest that AID, which is known to act only in the context of gene transcription and cause mutations for about 1500 bp downstream of TSS in B cell immunoglobulin genes (12), does not bind to actively transcribed regions or R-loop containing regions on its own. Much of the early biochemical work on AID that showed that mutagenicity of purified AID was greatly enhanced by *in vitro* transcription, used genes on plasmids as mutagenesis targets (34,62–64). Some of these systems lacked cellular processes of DNA replication and repair that create transient ssDNA, and also lacked topoisomerases that can create negatively supercoiled DNA. Consequently, they may have been strictly dependent on transcriptional pausing to create ssDNA substrates for AID (65). It is likely that in B cells the dependence on transcription is caused by the interactions of AID with proteins associated with transcription such as RNA polymerase II (66) and Spt5 (67) that are missing from *E. coli*.

In *E. coli*, about half the genes are transcribed in the same direction as replication fork movement while the rest are transcribed in the opposite orientation and we wondered whether the direction of transcription of genes could affect the observed deamination bias in favor of LGST. To evaluate this possibility, genes were divided into four categories depending on their presence in the left or right replichore, and their direction of transcription relative to replication (Supplementary Figure S8). While this analysis shows that there was a slightly higher bias in favor C to T changes in LGST when there was a replication-transcription conflict, this difference was not statistically significant (Supplementary Figure S8).

Only two of the uracilation peaks created by AID.cry and A3A overlap, and these peaks overlap TSS for the genes and a FIS-binding site about 70 nt upstream the TSS (Figure 2B). Mutational and computational studies of one of these operons, *leuQ*-*leuP*-*leuV*, have shown that binding of FIS upstream of the TSS causes this region to become more negatively supercoiled and reduces the amount of free energy needed to induce strand separation at the promoter and the TSS (68). This has been referred to as stress-induced duplex destabilization and has been implicated in a number of regulatory processes in *E. coli* (69). FIS also plays a structural role in the organization of supercoiled domains in the *E. coli* genome helping create what have come to be known as topologically associated domains or TADs (70,71). The local negative supercoiling causes the GC-rich DNA upstream of TSS to open up and promotes the transition of the transcription initiation complex from the closed to an open form. Although homologs of FIS have not been described in mammalian cells, studies in yeast have shown that there are more clustered mutations caused by AID/APOBECs in the TSS or the promoter than the gene body (61,72). These investigators implicated a transcriptional co-activator, Sub1, in this process (72).

There are interesting parallels between these observations and recent reports regarding the chromatin structural determinants involved in AID targeting. Senigl *et al* have shown the susceptibility of a region to SHM is correlated with the binding of cohesin-loading factor Nipped-B-like, NIPBL, and the presence of enhancer elements called diversification activators, DIVACs (73). They propose that binding of NIPBL promotes high levels of chromatin loop extrusion creating TADs, which can be targeted by AID if DIVACs are present within them. NIPBL has been reported to interact with the Mediator complex that regulates transcription initiation (74,75) and Senigl *et al.* suggest that the concerted action of NIPBL and DIVACs increases the rate of transcription initiation of genes within the TADs, but also promotes RNA polymerase II to stall more frequently (73,76). The parallels between the actions of FIS and NIPBL suggest that the DIVAC enhancer-binding proteins may help the TADs become negatively supercoiled (77,78) which helps binding of transcription factors that create pre-initiation complex (79,80) or promotes the formation of DNA structures such as R-loops favored by AID (31,32,37,81–83).

### Differences between A3A and AID.cry uracilomes

There were two major differences in the patterns of uracilation in genomes of cells expressing A3A and AID.cry. One striking difference between the two uracilomes is their relationship with the two strands in a replication fork. We and others have shown that several human APOBEC3 enzymes deaminate cytosines in the LGST much more frequently than in the LDST and that this was true in *E. coli* (21,22), yeast (20) or human cancer genomes (24,25). We showed here that AID.cry targets cytosines in the LGST only slightly more frequently than LDST (Figure 3A).

The second major difference between the two uracilomes was the preference (or the lack thereof) for cytosines in hairpin loops. While cytosines in predicted hairpin loops were deaminated by A3A four times more frequently than those in non-hairpin sequences, these two frequencies were about the same for AID.cry and the same pattern was found for the EV samples (Figure 3B). This lack of preference of AID for cytosines in hairpins is consistent with not only biochemical studies of purified AID (35), but also the crystal structure of AID.cry (37). Although that structure lacked bound DNA, Qiao *et al.* pointed out that the putative DNA binding channel in AID.cry is substantially different from the channel in A3A and is unlikely to accommodate a U-shaped substrate (37).

**Figure 6.**
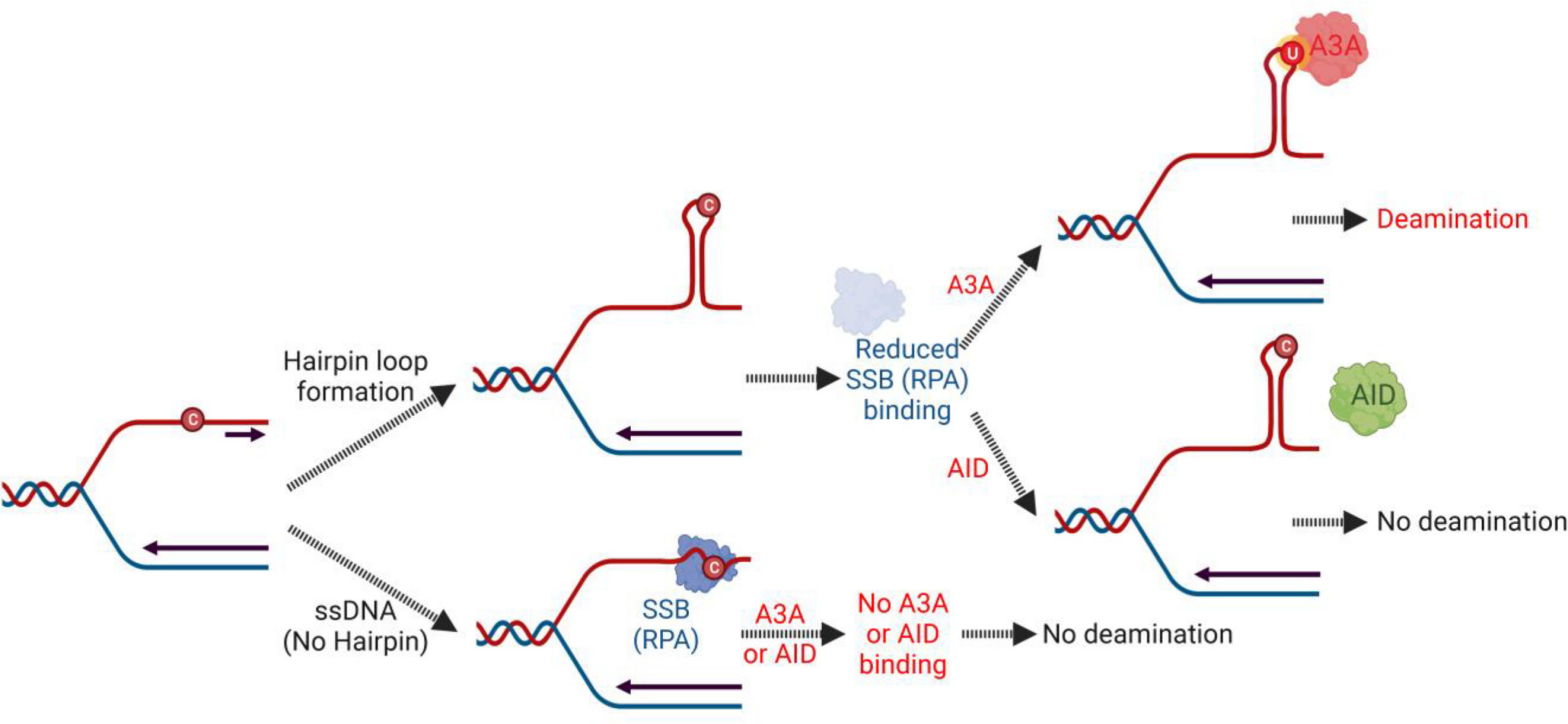
A model for the protection of replication fork DNA from AID. Proposed relationship between hairpin loop formation, binding of SSB (or RPA in eukaryotic cells) to single-stranded (ssDNA) and protection of cytosines in the LGST against deamination by AID is shown. The LGST within replication forks contains ssDNA that may be unstructured (lower path) or can form a secondary structure such a hairpin loop (upper path). The unstructured DNA will be bound by SSB in *E. coli* (or RPA) preventing both A3A and AID from deaminating cytosines in this DNA (lower path). However, if the cytosine lies within a potential loop of a hairpin, this will reduce the binding of SSB (RPA) to this DNA. This exposed cytosine within the loop will be a good substrate for A3A, but not for AID (upper path). In both the upper and lower paths, the cytosines in ssDNA at replication forks will be protected against deamination by AID.

The relative lack of preference of AID for cytosines in hairpin loops may explain its inability to preferentially attack the LGST. The ssDNA at the replication forks in *E. coli* is protected by SSB against nucleases and 35–65 nt of ssDNA is wrapped around this multimeric protein (84). It is likely that strong DNA hairpin structures inhibit the wrapping of DNA around SSB and this makes the cytosines in the loops susceptible to deamination by A3A, but not by AID. The mammalian equivalent of SSB, RPA, binds poorly to hairpin loops (26,29) and is much less efficient at protecting cytosines in hairpin loops against deamination by A3A than in unstructured ssDNA (26). As a consequence, A3A preferentially deaminates cytosines in the hairpins that form within the ssDNA of LGST, but AID is unable to do so (Figure 6).

### A model for how AID may avoid deaminating cytosines at replication forks

The lack of preference shown by AID for hairpin loops and LGST makes biological sense because the primary function of AID is to act on *IG* genes in the human genome. Unlike the APOBEC3s, which act on infecting viruses and transposing retroelements, AID acts on *IG* genes during B cell development (5). Hence it is important for the B cell to avoid AID from acting on the rest of the genome. This may be accomplished in part by entry of AID into nuclei only during the breakdown of the nuclear membrane during late G2 and subsequent export out of the nucleus (85). This assures that AID is present in the nuclei only during early G1 and is not retained during the S phase (85). However, others have reported that AID may be actively transported into the nucleus using a nuclear localization signal recognized by karyopherins and this may occur at other stages of the cell cycle (2,86). It has also been suggested that AID enters the nuclei in short pulses and is then exported out (87). Thus, it is possible that some AID resides in the nucleus outside G1. The data presented here suggests a back-up mechanism for the protection of the ssDNA at replication forks from AID. The binding of RPA to ssDNA within the replication forks and the inability of AID to deaminate most cytosines in hairpin loops, protects the largest source of ssDNA during the S-phase, DNA replication (Figure 6). Thus, the differences between AID and APOBEC3s in their preferences for secondary structure is rooted in their different biological roles.

In summary, we have shown here that while the AID and A3A uracilomes have some common features such as preference for tRNA genes and TSS, they have substantial differences in terms of replicative strand bias and DNA secondary structure preferences. Their similarities reflect an affinity for intrinsically single-stranded regions of the genomes, while their differences may reflect structural differences within the proteins that make them better suited for their respective biological functions.

## DATA AVAILABILITY

All the UPD-Seq raw sequencing data is available at NCBI Sequence Read Archive under the BioProject IDs PRJNA801888 and PRJNA448166.

The list of fastq files and their accession numbers are provided in Supplementary Table S3.

Functions or scripts used for the analysis are uploaded to a GitHub repository at:

https://github.com/rayanramin/AID.cry-UPDSeq.

Hairpin Survey Analysis of BH214, its genomic annotations, NDC2 values behind Figure S3 have been deposited in Zenodo:

https://zenodo.org/record/5979670#.Yf7uai-B2Lc.

## Supplementary Material

gkac296_Supplemental_Files
