# Supplementary material for: Human activation-induced deaminase lacks strong replicative strand bias or preference for cytosines in hairpin loops
Source: Nucleic Acids Res. 2022 May 7;50(9):5145–57. doi: 10.1093/nar/gkac296 (PMC9122604; doi:10.1093/nar/gkac296)
Supplement: gkac296_Supplemental_Files [file gkac296_supplemental_files.zip › Supplementary Figures.pdf]

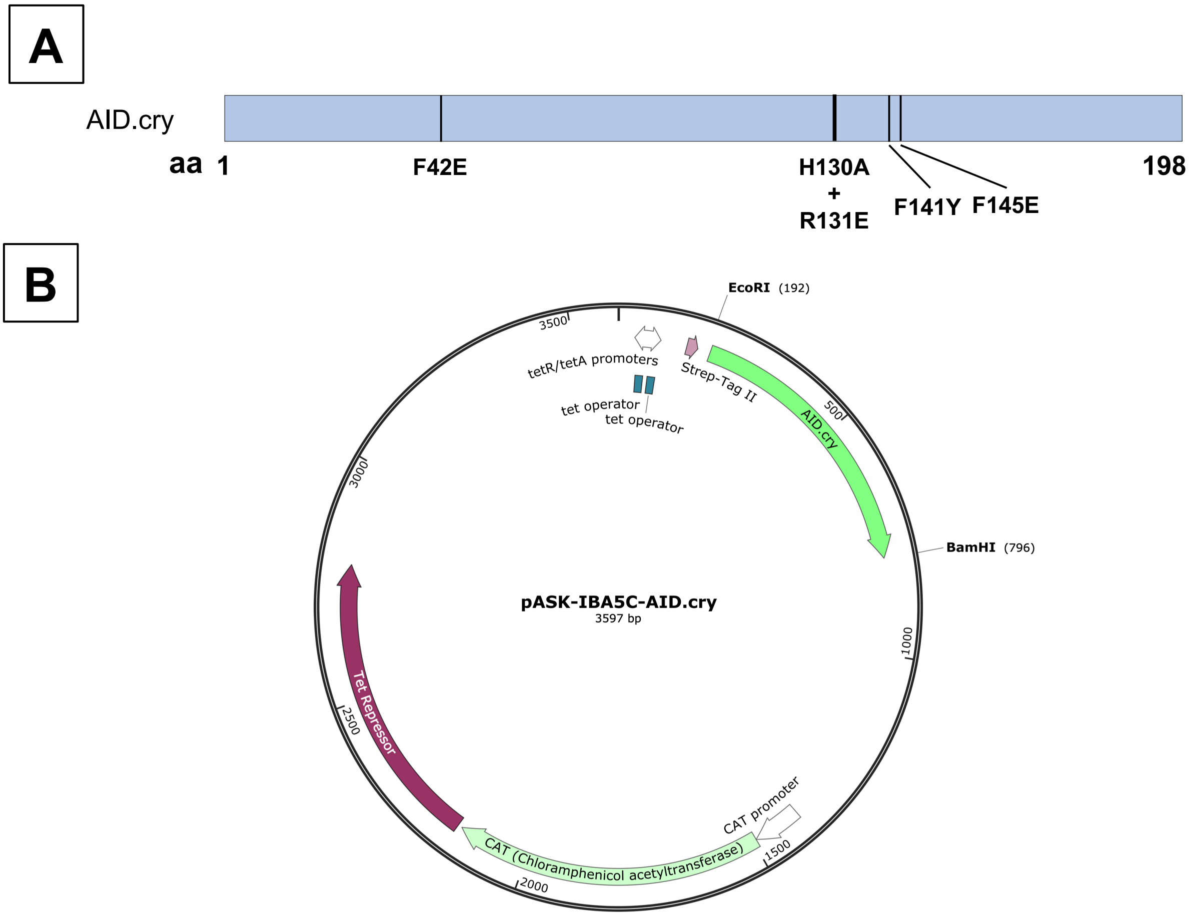

Figure S1. Schematic representation of the AID.cry gene and its *E. coli* expression plasmid. A. AID.cry is full-length human AID with the indicated mutations. **B.** Map of pASK-IBA5C plasmid with the AID.cry gene shown in green.

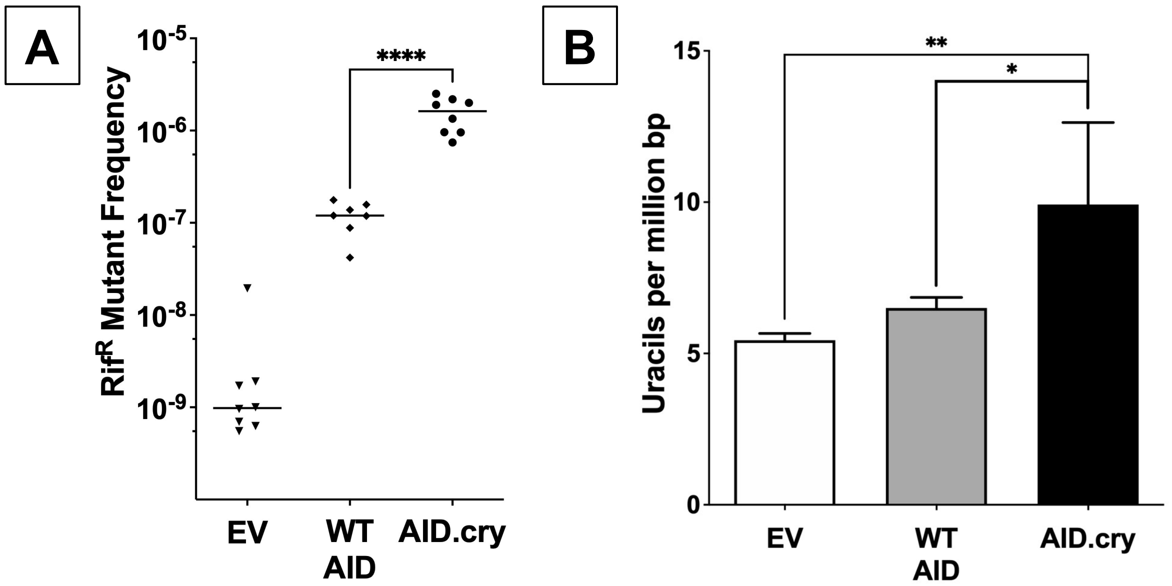

Figure S2: Mutations and uracils created by AID in repair-defective *E. coli.* Data for WT AID, AID.cry and EV (empty vector) are shown. **A**. Rifampicin-resistant mutant frequencies from 8 independent cultures are shown with the median value indicated by a horizontal line. **B**. Quantification of genomic uracils. Uracils levels in DNA from six independent experiments are shown with mean and standard deviation. P-values are shown as * for P ≤ 0.05; ** for P ≤ 0.01; *** for P ≤ 0.001 and **** for P ≤ 0.0001.

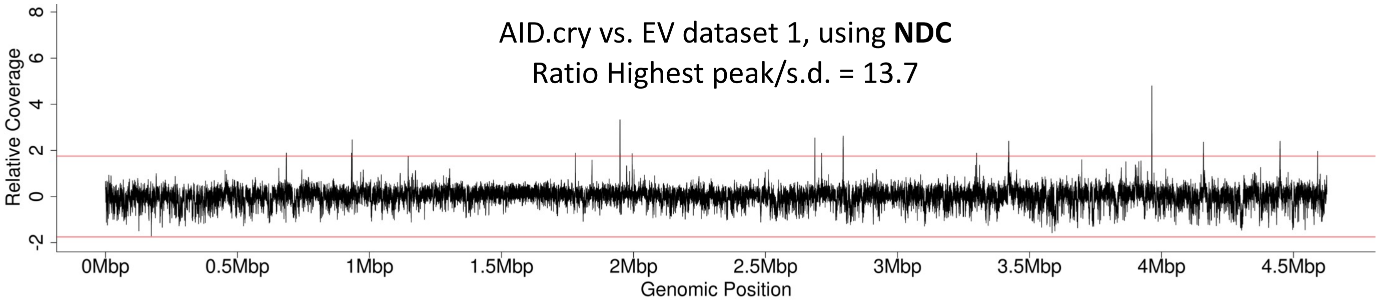

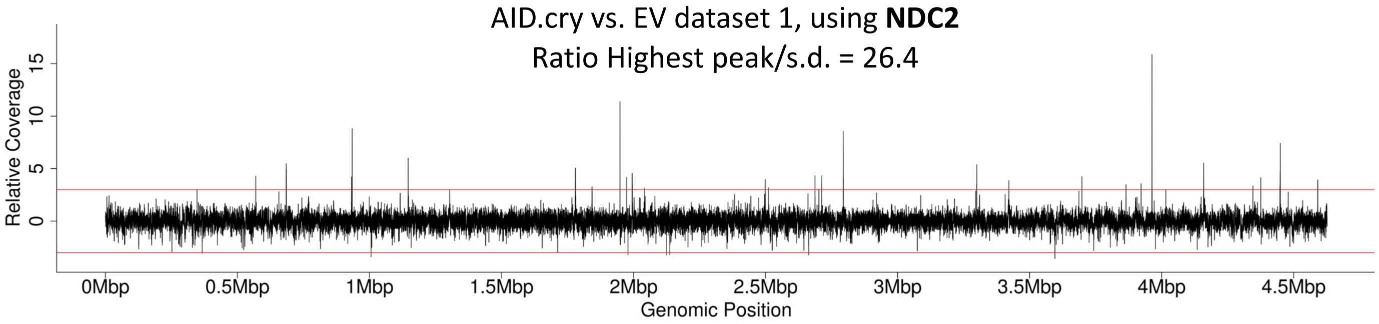

Figure S3: Analysis of UPD-seq data for AID.cry using NDC and NDC2. The red lines indicate mean coverage ± 5x(standard deviation).

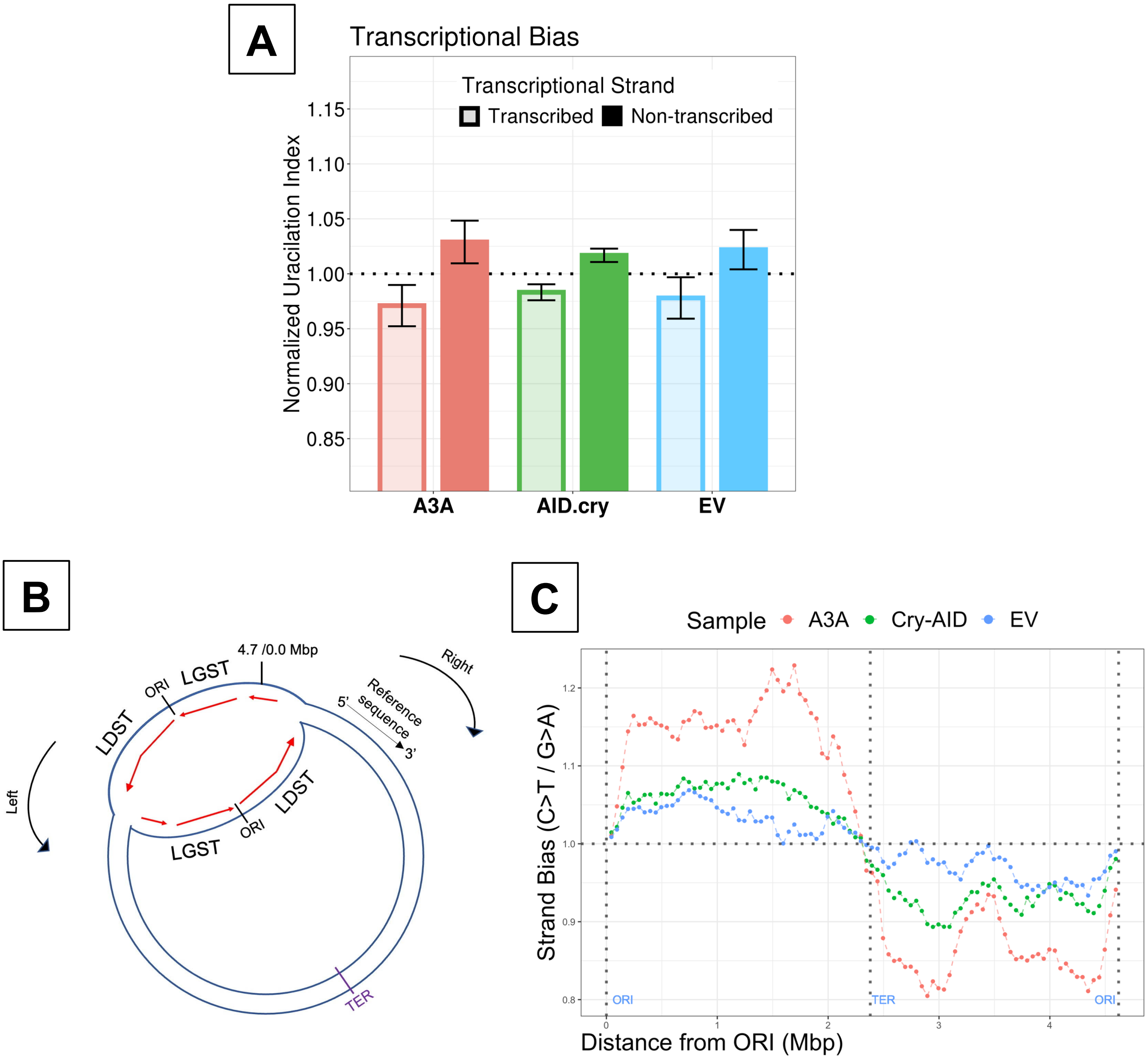

Supplementary Fig. S4. Strand bias in uracilation by A3A and AID.cry.
A. Transcriptional strand Bias in A3A,AID.cry and EV, unpaired t-test comparing A3A to EV and AID.cry to EV results in non-significant p-values. **B.** Schematic representation of the two replichores of *E. coli*. The directionality of the reference sequence is shown and the overall direction of replication in each replichore is indicated by arrows labelled “Right” and “Left”. The origin of replication (ORI), termination site for replication (TER), LGST and LDST are marked. **C.** Strand Bias over the genome. The ratio of (C-to-T/G-to-A) base changes are calculated at all cytosines in overlapping bins of 500kb, using the UPD-Seq data of different samples.

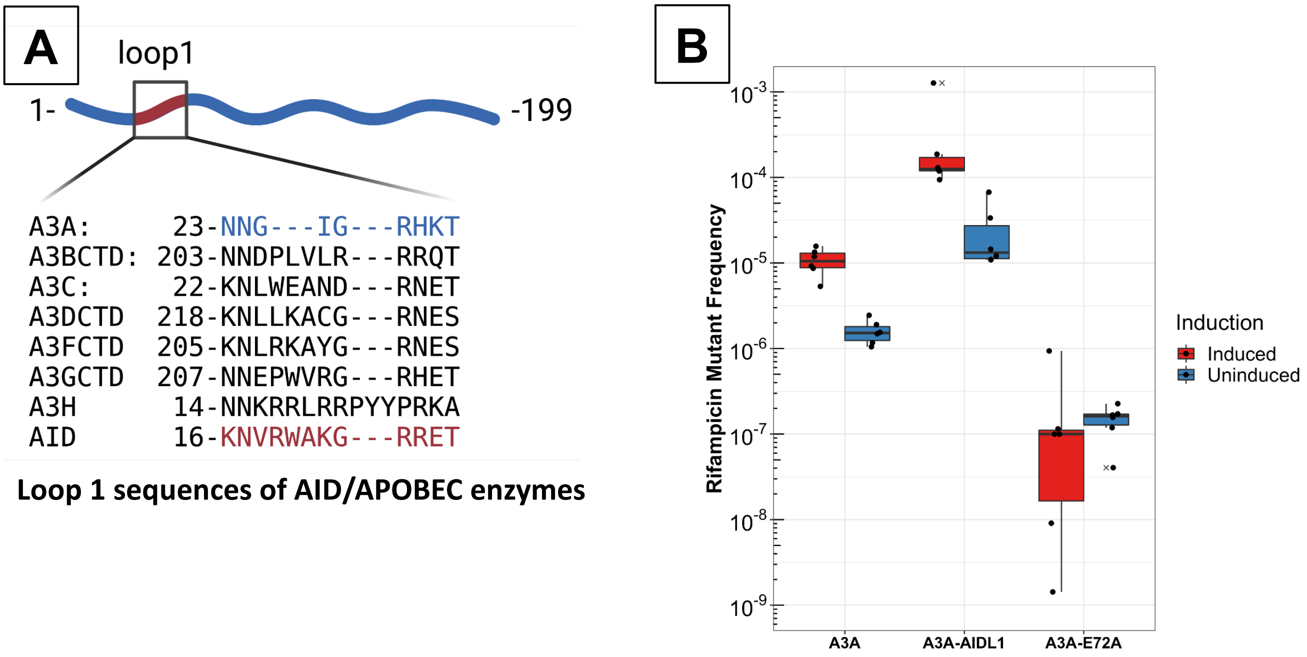

Supplementary Fig. S5. Structure and properties of A3A-AIDL1 mutant. **A.** Amino acid sequences of loop1 of AID/APOBEC enzymes. In A3A-AIDL1 chimeric enzyme, loop1 sequence of A3A is swapped with loop1 sequence of AID; **B.** Rifampicin mutant frequencies of BH214 cells transformed with inducible plasmids expressing A3A, A3A-AIDL1 or A3A-E72A. Red bars- induced cells; Blue bars- uninduced cells.

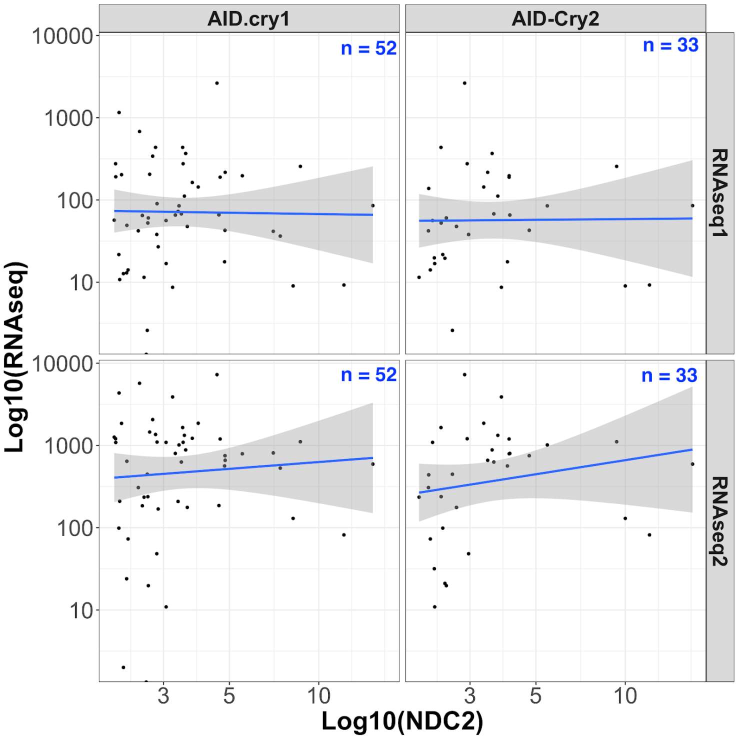

Supplementary Fig. S6. Modest correlation between high NDC2 values and transcription.. Only genes where integral of NDC2 values (normalized by gene length) is more than 2 are shown. These are the genes where UPD-Seq coverage in the treatment samples (AID.cry) are on average twice the control samples (EV).

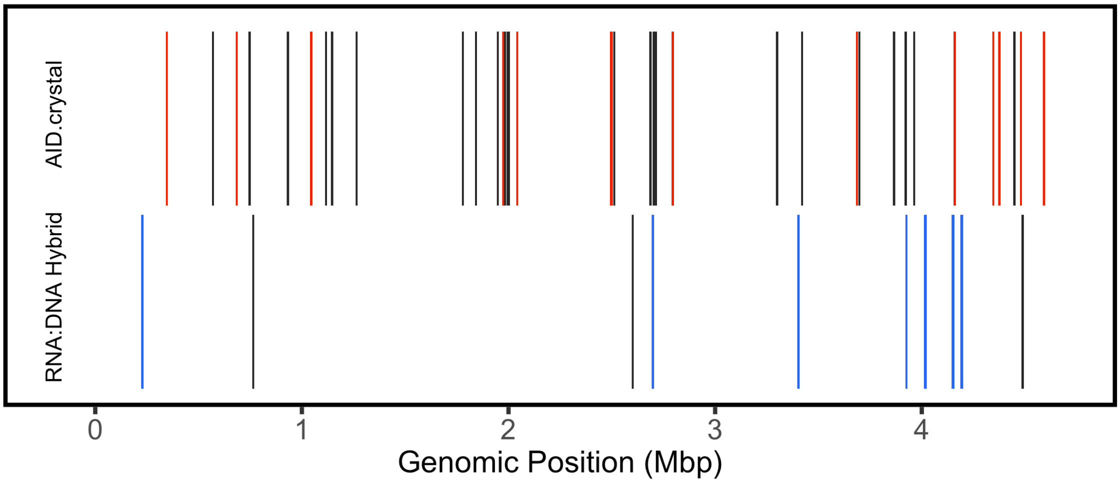

Supplementary Fig. S7. Comparison of AID.cry uracilation peaks with peaks from S9.6 antibody pull-down. Genomic positions of combined uracilation peaks from the two AID.cry datasets and a S9.6 antibody ChIP-seq experiment showing RNA:DNA hybrids are shown as vertical bars. Peaks overlapping with tRNA, rRNA and protein-coding genes are respectively shown in red, blue and black. The S9.6 data are from Sakhtemani *et al* (2019) *J. Biol. Chem.* 294(41):15037-15051.

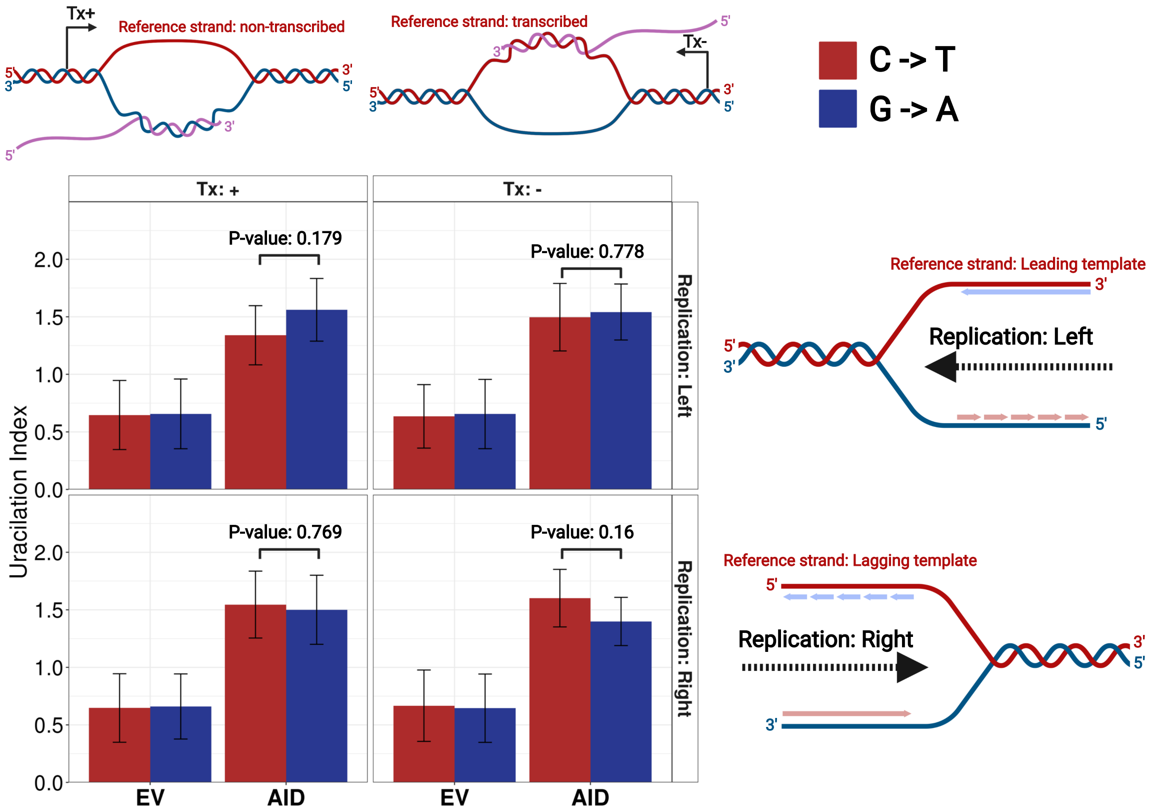

Supplementary Figure S8. Possible effects of the relative orientation of replication and transcription of genes on deamination by AID. The direction of replication is shown on the right of the bar graph, while the direction of transcription is shown above the graph. Reference DNA sequence strand, leading strand synthesis (continuous blue or red line), lagging strand synthesis (broken blue or red line), RNA transcript and transcription start sites are also marked. Red bars represent C to T changes, while blue bars represent G to A changes in the sequence.
