# Supplementary material for: Human activation-induced deaminase lacks strong replicative strand bias or preference for cytosines in hairpin loops
Source: Nucleic Acids Res. 2022 May 7;50(9):5145–57. doi: 10.1093/nar/gkac296 (PMC9122604; doi:10.1093/nar/gkac296)
Supplement: gkac296_Supplemental_Files [file gkac296_supplemental_files.zip › Supplementary Table Legends.docx]

**Supplementary Table Legends**

**Supplementary Table S1**

Primers used for cloning and site-directed mutagenesis.

**Supplementary Table S2**

Genes overlapping uracilation peaks. tRNA genes are shown in red.

**Supplementary Table S3**

The list of fastq files and their accession numbers.
